# Possible assistive technology solutions for people with moderate to severe/profound intellectual and multiple disabilities: considerations on their function and long-term role

**DOI:** 10.1080/20473869.2024.2303532

**Published:** 2024-01-16

**Authors:** Giulio E. Lancioni, Nirbhay N. Singh, Mark F. O’Reilly, Jeff Sigafoos

**Affiliations:** 1 Lega F. D’Oro Research Center, Osimo, Italy; 2 Department of Psychiatry and Health Behavior, Augusta University, Augusta, GA, USA; 3 College of Education, University of TX at Austin, Austin, TX, USA; 4 School of Education, Victoria University of Wellington, Wellington, New Zealand

**Keywords:** technology, intellectual disability, multiple disabilities, technology support, technology fading

## Abstract

The paper presents several assistive technology solutions that may be used to help people with moderate to severe/profound intellectual and multiple disabilities reach relevant goals such as strengthening object-manipulation and ambulation responses and carrying out multistep tasks. For each of the technology solutions, the paper examines (a) the specific function (i.e. the way it helps people achieve the goal targeted) and (b) the possible role over time (i.e. whether it might serve as a temporary or permanent form of support). The basic functions of the assistive technology solutions presented entail: increasing people’s motivation, supplementing people’s cognition (working memory), and bridging the gap between people’s motor, sensory/orientation, and verbal functioning and the functioning level required to achieve the goals targeted. Based on the knowledge available to date, the role of the technology solutions over time may be considered: (a) modifiable, with the possibility of reducing or even completely fading out the use of such solutions, when their function is to increase people’s motivation or support people’s cognitive/memory performance, and (b) practically unalterable when their function is to bridge the gap between people’s limited sensory, motor, and verbal skills and the skill level required to reach a relevant goal.

## Introduction

Assistive technology is being increasingly employed in intervention programs for people (i.e. children, adolescents, and adults) with intellectual and multiple disabilities (Boot *et al.* 2017, Rasouli *et al.* 2021, Sulaimani and Bagadood 2023). Its employment is basically intended to support people’s access to their environment and promote their level of self-determination and constructive engagement (e.g. engagement in simple motor responses, complex activities, and communication) with affordable staff time costs (Abdi *et al.* 2021, Mensah-Gourmel *et al.* 2023). The technology employed is typically adapted to the people’s condition (i.e. types and levels of disability) and aimed at helping them achieve goals that are relevant for advancing their status and in line with the education, rehabilitation and care plans of their daily contexts (Halvorsrud *et al.* 2023, Rasouli *et al.* 2021).

Among the main goals pursued with people with severe or profound intellectual and multiple disabilities one may include the independent performance of (a) object-manipulation and ambulation responses, (b) basic choice and communication/request responses, and (c) simple occupational activities involving object use and mobility within a daily context (e.g. collecting, transporting and putting away objects) (Lancioni *et al.* 2009a, 2022a, 2023b, Stasolla *et al.* 2017, 2019). Among the goals pursued with people with moderate intellectual and multiple disabilities, one may include the independent performance of multistep tasks (e.g. vocational or domestic tasks) (Desideri *et al.* 2021, Golisz *et al.* 2018, Lin *et al.* 2018) as well as the combination of multistep tasks with leisure engagement and communication with distant partners (Lancioni *et al.* 2023a). The technology required to support the achievement of the different goals can vary substantially with regard to the elements/components involved and their working characteristics (Fernández-Batanero *et al.* 2020, Mensah-Gourmel *et al.* 2023).

This paper presents a number of assistive technology solutions that may be used (i.e. as indicated by the literature available) to help people achieve the goals mentioned above. For each technology solution, the paper examines the specific function (i.e. the way it helps people achieve the goal targeted) and the possible role over time. With regard to the role issue, the question asked is whether the technology solution (i.e. based on the function it has) can be gradually faded out without performance disruption or whether it remains critical for ensuring performance maintenance and thus no fading is possible. The rehabilitation goals and the technology solutions described in the paper are a convenience sample of those available in the literature. The paper in fact is not intended to provide a comprehensive review of those goals and solutions, but to present some of them (used with people with intellectual and other disabilities) as examples for examining the aforementioned issues of technology solutions’ function and role over time. Examining these issues was thought to be an informative operation that may have practical implications for rehabilitation and care contexts using technology solutions in their daily programs.

## Technology solutions: function and long-term role

### Example 1: severe/profound intellectual disability and sensory or motor impairments

People with severe/profound intellectual disability and sensory or motor impairments may be largely passive and often prone to engage in stereotyped behavior and have minimal or no interest (motivation) to engage in forms of functional activity such as object manipulation and ambulation (Maes *et al.* 2021, Nijs and Maes 2021, Stasolla *et al.* 2017). A plausible technology solution for supporting object manipulation and ambulation responses may entail sensors (e.g. optic and touch devices) interfaced to a computer, tablet or smartphone fitted with specific software or commercial applications (Lancioni *et al.* 2022a, Stasolla *et al.* 2019, Tam *et al.* 2011). The sensors would be set up for monitoring the occurrence of the responses. The computer and tablet/smartphone would be programmed to provide brief periods of preferred stimulation in relation to (contingent on) the responses (Lancioni *et al.* 2022b, Stasolla *et al.* 2017). In essence, the technology in this situation would serve to make the responses meaningful to people (i.e. by allowing people to use those responses as means for achieving preferred stimulation; Briggs *et al.* 2023).

In light of the above, it seems clear that the basic function of the aforementioned technology solution is to motivate the people’s performance of object manipulation and ambulation responses (Stasolla *et al.* 2017). Technology-aided intervention studies conducted to help people perform these responses have not provided evidence as to whether the role of the technology solution might be curtailed over time (i.e. with a parallel reduction in the frequency of stimulation events available for the responses). In contrast with this lack of direct evidence, one can find a large body of literature concerning other populations and responses that shows the possibility of lowering (thinning) the level of stimulation and using intermittent stimulation without performance disruption (Briggs *et al.* 2023, Kranak and Brown 2023, Pierce and Cheney 2017). Based on this literature, one can argue that following the strengthening of object manipulation and ambulation responses, the technology might be set to provide stimulation after two or more of those responses (rather than after each one of them; Pierce and Cheney 2017, Stasolla *et al.* 2018). Stimulation thinning might be extended even further with some people (Briggs *et al.* 2023). In a few cases, eventually, it might be possible to postpone the stimulation to the end of brief ambulation or object manipulation periods without performance declines. In these cases, one would consider the use of the technology setup as initially designed no longer necessary.

### Example 2: severe/profound intellectual and multiple disabilities

People with severe/profound intellectual and multiple disabilities are often unable to exhibit any specific interaction with their environment and to make choices and requests due to their limited motor repertoire, lack of speech, and tendency to be passive (Lancioni *et al.* 2022a, Nijs and Maes 2021, Stasolla *et al.* 2019). A plausible technology solution for supporting choice and request responses may entail different (e.g. pressure, touch, and optic) sensors that are interfaced to a computer or tablet/smartphone fitted with specific software or commercial applications (Lancioni *et al.* 2009a, 2009b, 2022a, Stasolla *et al.* 2019). The sensors would be adapted to the people’s motor conditions and thus could be triggered through a variety of responses, which may also include small (simple) movements such as head turning, eyelid prolonged closures, and hand or finger movements (Lancioni *et al.* 2009a, 2009b, 2022a). Each sensor would be linked to a specific event (e.g. a specific type of stimulation, a call for caregiver attention, or a verbal request for a preferred activity). With this technology setup, people would be able to choose which of two or more particular events they want to access or request by simply triggering the sensor specifically linked to that event (Stasolla *et al.* 2019).

The two main points that make the technology solution functional are: (a) the availability of sensors that allow people to successfully use small non-verbal responses to express their choices and to verbalize their requests, and (b) the possibility of having their choices followed by preferred stimulation events and their requests followed by caregiver attention or other forms of preferred activity. Any decision to fade out the technology following people’s performance progress (i.e. with technology support) would be justifiable only if people (a) managed to acquire (i.e. during the time in which the technology was in use) the motor skills required to independently activate environmental stimulus sources (e.g. a music playing device) and the verbal skills to make requests or (b) are considered capable of acquiring such skills. Each of these prospects may be deemed rather unrealistic in light of two specific facts. First, no study has reported any such development followed by a fading out of the technology support. Second, pervasive motor impairments and lack of speech due to severe cerebropathy cannot be considered a transient condition and thus no significant developments (improvements) would be expected in such condition (Danker *et al.* 2023, Hoyle *et al.* 2020, Medforth and Boyle 2023, Smith *et al.* 2021). Given the above, the technology would need to remain in use and serve as a permanent prosthesis capable of bridging the gap between the people’s skill (response) level and the functioning level required for accessing and choosing between environmental events and making successful requests.

### Example 3: severe intellectual disability and visual impairments

People with severe intellectual disability and visual impairments may have difficulties finding objects in different areas of their daily context and in transporting and putting away those objects because of low motivation to be active, spatial orientation problems, and confusion as to when and where to take and put away objects (Dijkhuizen *et al.* 2016, Lancioni *et al.* 2017, Parker 2009). A technology solution for supporting mobility and occupational engagement (e.g. collecting, transporting and putting away objects) may involve motion sensors, a computer or tablet/smartphone with specific software or commercial applications, and mini speakers (Lancioni *et al.* 2023b). The motion sensors would be interfaced to the computer or tablet/smartphone and would serve to determine the people’s arrival at and/or departure from areas (working stations) located within their daily context. The computer or tablet/smartphone could be set up to present (a) auditory cues that would serve as spatial orientation means, as encouragements/prompts, and as specific object-use instructions to help people move to the destinations and collect and put away objects, and (b) preferred stimulation in relation to people’s responses of collecting and putting away objects. A mini speaker would be available at each destination and would serve to deliver the computer or tablet/smartphone’s auditory cues when people are to reach that destination and computer or tablet/smartphone’s preferred stimulation as soon as people have reached that destination.

As alleged above, the function of this technology solution is to motivate people to be constructively busy and provide them with the support needed to circumvent their problems in finding/locating, collecting, and putting away objects (Dijkhuizen *et al.* 2016, Lancioni *et al.* 2023b). No specific research evidence is available as to whether this technology solution may be temporary or rather needs to be permanent. At present, the most realistic hypothesis is that its support will tend to remain necessary over time for a fairly large group of people with severe intellectual disability and visual impairments based on two reasons. First, these people may continue to have difficulties in spatial orientation even within relatively small occupational contexts (Dalyot and Cohen 2022, Lancioni *et al.* 2017, Nair *et al.* 2022, Parker 2009). Second, the same people may also have difficulties in the correct use of objects (e.g. in determining where to take and where to put away objects) (Dijkhuizen *et al.* 2016, Lancioni *et al.* 2017). One change that might be realistic concerns the technology aspects designed to motivate people to be active. That is, a certain (gradual) reduction in the amount of preferred stimulation delivered for collecting and putting away objects would be expected to be possible without a loss of engagement motivation (see Example 1).

### Example 4: moderate or moderate-to-severe intellectual disability

People with moderate or moderate-to-severe intellectual disability often have problems in carrying out multistep tasks because of their inability to remember the steps involved or the correct sequence of those steps (Desideri *et al.* 2021, Randall *et al.* 2020). The technology for promoting the independent performance of multistep tasks may involve a computer or tablet/smartphone programmed to contain and present the instructions for the single steps of the tasks (Desideri *et al.* 2021, Muharib *et al.* 2022). The instructions may entail pictorial images representing the material/objects to be used for the single steps, verbal guidance (description of the actions required) for the performance of the single steps as well as mini videos illustrating the performance of the various steps (Desideri *et al.* 2021, Randall *et al.* 2020). Those instructions may be presented by the computer or tablet/smartphone (a) automatically at preset time intervals or (b) following a simple people’s operation such as touching a specific area of the tablet screen (Desideri *et al.* 2021). In some cases, the instructions may be automatically presented by the computer or tablet/smartphone in close time connection with the people’s performance of the task steps (Lin *et al.* 2018, Mihailidis *et al.* 2016). Such an arrangement ensures that the instruction for a new step always occurs immediately after the completion of the previous step. In those cases, the technology also involves the use of sensors monitoring the people’s step performance so as to control the timing of the instructions (Lancioni *et al.* 2021, Mihailidis *et al.* 2016).

The technology solution options presented above to help people perform multistep tasks are mainly intended as a form of support/supplement for the people’s cognitive (working memory) domain. The role of the technology solution used may change over time depending upon the people’s level of intellectual disability, the number of tasks available, and the number of steps involved in the tasks. When only one or very few tasks with relatively few steps are used and the people’s level of intellectual disability is in the high moderate range (or in the moderate-to-mild range), there is a real chance that the role of the technology solution may be gradually curbed (Sigafoos *et al.* 2007, Wu *et al.* 2016). This may involve the complete withdrawal of the technology support or the technology’s presentation of the instructions for the task steps in chunks rather than individually (Sigafoos *et al.* 2007). The chunks, moreover, may become gradually bigger (i.e. involving a longer string of instructions) so as to increase the people’s independence level (Sigafoos *et al.* 2007, Shepley 2017, Wu *et al.* 2016). A different picture may emerge when multiple tasks are available that include large numbers of steps (Desideri *et al.* 2021). In those cases, the more realistic prospect is not the withdrawal of the technology, but a new setup of it that would allow the presentation of the instructions in chunks with their dimensions tailored to the capacity of the single individuals using them (Sigafoos *et al.* 2007, Shepley 2017).

### Example 5: moderate intellectual disability, lack of (clear) speech, and motor or sensory-motor impairments

People with moderate intellectual disability, lack of (clear) speech, and motor or sensory-motor impairments may have serious problems in carrying out multistep tasks (see above). They may also have serious difficulties in accessing leisure events (i.e. in using leisure-related tools such as television and computers) or communicating with distant partners (i.e. in using telephone or other communication devices) (Chan *et al.* 2013, Darcy *et al.* 2017, Lancioni *et al.* 2023c). The technology for supporting the combination of multistep tasks with access to leisure and communication with distant partners may involve a smartphone fitted with Internet connection, Google assistant, a SIM card, a commercial (programmable control) application, mini recording devices, and mini speakers (Lancioni *et al.* 2023a). The application may be programmed to make the smartphone alternate time periods in which people can choose between accessing preferred stimulation events (e.g. music) and making voice or video calls with distant partners, with time periods in which people receive instructions for the performance of multistep tasks. The choice between leisure (preferred stimulation events) and communication (calls to distant partners) can be operated *via* the mini recording devices each of which would have a recorded message (i.e. either the request for a leisure event or the request for a telephone call with a specific partner). When activated by a simple hand-pressure response, a device would utter the recorded message. This message would trigger the Google assistant and, through the control (programmed) application, regulate the smartphone’s presentation of a leisure event or the start of a telephone call with a distant partner.

The function of this technology solution is twofold. First, it circumvents the task performance problems by providing people with task step instructions. Second, it enables people to control the smartphone or tablet through simple hand pressure responses (i.e. with no need of using elaborate visuo-motor operations on the smartphone/tablet and of producing verbal utterances recognizable by the smartphone/tablet). Given that the sensory, motor, and verbal condition of these people (particularly if adults) cannot be expected to significantly change over time (Halvorsrud *et al.* 2023, Hoyle *et al.* 2020, Nakayama 2023, Smith *et al.* 2021), it would be practically impossible for them to acquire the ability to perform elaborate visuo-motor operations or to produce intelligible verbal utterances (i.e. ability required in the absence of the technology solution). On this basis, one can argue that the technology solution will continue to be necessary at least for accessing leisure and communicating with distant partners. With regard to the performance of multistep tasks, one could reiterate the points expressed in *Example* 4.

## Discussion

This paper has presented a number of technology solutions used to help people with intellectual and multiple disabilities reach relevant rehabilitation goals. For each technology solution, the paper examined the specific function (contribution) and the likely role over time, in an attempt to provide clarifications that could eventually be helpful for rehabilitation and care contexts using those technologies within daily programs. The functions identified for the technology solutions presented entail: (a) increasing people’s motivation to perform different types of useful, adaptive responses, (b) supporting/supplementing the people’s cognitive (working memory) domain, and (c) bridging the gap between the people’s motor, sensory/orientation, and verbal functioning levels and the functioning skills required to achieve the goals targeted.

With regard to the role of the technology solutions over time, a number of considerations were made based on the literature evidence available to date. First, technology solutions used to promote people’s motivation may be gradually (partially) faded out over time (Briggs *et al.* 2023, Pierce and Cheney 2017). In essence, a successful initial use of the technology with consequent response strengthening may be followed by a thinning of the stimulation events that the technology provides and occasionally it may even lead to brief periods of successful response engagement without the technology and related stimulation deliveries (Briggs *et al.* 2023, Kranak and Brown 2023).

Second, technology solutions used as memory supplement to support people’s performance sequences such as multistep tasks may also have their role revised (reduced) over time. For example, some people may acquire the ability to remember the task steps and their sequence and thus become independent of the technology’s instruction deliveries (i.e. when only few and relatively brief tasks are available; Sigafoos *et al.* 2007). Other people may (a) become capable of performing the tasks with the step instructions provided in chunks rather than individually and, through this development, (b) point to the fact that the technology’s role is being curtailed (Shepley 2017, Wu *et al.* 2016).

Third, technology solutions used to bridge the gap between people’s limited sensory, motor or verbal skills and the skill level required to achieve the goal targeted for them are typically expected to have a long-term role (i.e. to serve as prosthetic tools) (Johnson *et al.* 2023, Lancioni *et al.* 2009b, 2022b, Nair *et al.* 2022). The sensory, motor or verbal condition of most of these people in fact (particularly adults) cannot be considered transient. This implies that they (a) are unlikely to have a clear improvement of their skill their sensory, motor or verbal skill levels (i.e. to reach the levels required by the goals targeted), and consequently (b) will continue to rely on the support provided by the technology for maintaining their performance (Howard *et al.* 2022, Hoyle *et al.* 2020, Nakayama 2023, Smith *et al.* 2021).

In conclusion, four basic issues may be pointed out for new studies to address. The first issue concerns the need to extend the preliminary evaluation carried out in this paper to other technology solutions and rehabilitation goals so as to have a more comprehensive and informative picture of what is available and how it can be arranged. The second issue concerns the need to clarify when and how far one can fade out the technology solutions aimed at promoting people’s motivation and supporting people’s cognitive (memory) functioning (Briggs *et al.* 2023, Shepley 2017, Stasolla *et al.* 2018, 2019). The third issue concerns the need to identify common daily situations in which technology solutions (a) serve to bridge serious gaps between the people’s ability and the skills level required to achieve important rehabilitation goals, and thus (b) are likely to become permanent prosthetic aides (Howard *et al.* 2022, Lancioni *et al.* 2023a, Nair *et al.* 2022, Smith *et al.* 2022). The fourth issue concerns the need to develop new technology solutions that are easily accessible, practical and unobtrusive, and friendly for the people involved and their staff/caregivers (Borg *et al.* 2023, Botelho 2021, Horton 2021). The demand for new solutions seems even greater in those situations in which reliance on technology is very likely to be long-term (Mishra *et al.* 2022).
